# Supplementary material for: A genome-wide association study of neutrophil count in individuals associated to an African continental ancestry group facilitates studies of malaria pathogenesis
Source: Hum Genomics. 2024 Mar 15;18:26. doi: 10.1186/s40246-024-00585-w (PMC10941368; doi:10.1186/s40246-024-00585-w)
Supplement: Supplementary file 1 — Additional file 1. Supplementary Methods on GWAS with references. [file 40246_2024_585_MOESM1_ESM.docx]

SUPPLEMENTARY METHODS

**Pre-GWAS investigative analyses**

Descriptive analyses of nc_log were performed. To study the amount of potential population admixture in the AFR_CAG that could affect the test statistics from a GWAS, an analysis was conducted in R on the Duffy SNP rs2814778 [1] for each Kpop. The preponderance of the Duffy SNP rs2814778 allele distribution was outlined in a PCA plot (PC1~PC2), and its association with nc_log in the AFR_CAG dataset was studied with and without PCs. GWAS are usually performed using individuals of a similar genetic background to avoid SNP-trait associations that are biased or are false-positives due to a confounding effect by ancestry [59]. However, even in white British individuals from UKBB, latent population structure can affect SNP effect sizes, which may not be completely removed by adjusting for PCs [60]. Nevertheless, we investigated the number of PCs that should be added into the GWAS to control for population structure. The Tracy-Widom statistic from the EIGENSOFT package [61] indicated over 100 significant PCs. However, there is no exact way to establish how many PCs should be added into a GWAS, although an excessive number of PCs can lead to a reduction in power, while too few might bias GWAS effect sizes due to residual population structure [48]. Previous studies have added 40 to 100 PCs in their UKBB GWA analyses [60,62,63], hence our inclusion of the first 100 PCs as covariates.

**SNPTEST and META GWAS**

To test whether the effect estimates from the BOLT-LMM GWAS were biased due to residual population structure that would characterise a population from the African CAG, a number of “sensitivity” GWAS were conducted. This was done with SNPTEST using a linear model algorithm [2,3], with 16 GWAS conducted as follows: 8 GWAS were run on each K-means cluster (Kpop) + the whole sample with the same parameters as in the BOLT-LMM run, and another 8 in the same manner, but with rs2814778 as an additional covariate. To minimise the chance of errors and to reduce the time needed to run each GWAS, a linear model was first conducted in R using the command “lm(nc_log ~ my_covariates)”. The residuals were then pulled with the “residuals()” function and 16 GWAS were run on AFR_CAG with SNPTEST. The Kpop GWAS were then meta-analysed with META [4] under an inverse-variance method based on a fixed-effects model. The result were two meta-analyses: one without accounting for the Duffy SNP rs2814778 called “META-WOD”, and one where the Duffy SNP was included as a covariate, called “META-WD”.

**Conditional & joint association analysis**

GCTA-COJO [5,6] was employed to identify independent signals from the BOLT-LMM GWAS, as well as detect any possible secondary signals arising from a stepwise selection model. SNPs which are close together are usually in LD i.e. their alleles are not random, but correlated [7]. Before running GCTA-COJO, genetic variants with an INFO score < 0.3 were filtered out of the AFR_CAG dataset with QCTOOL. PLINK was then used on this resulting output to filter out related individuals. Following this step, GCTA-COJO was run on the AFR_CAG filtered dataset to identify causal SNPs. These were referred to as “index” in the text. Plots similar to those generated by LocusZoom [8,9] were created in R with the “LocusZooms” package [10].

**Genomic inflation**

The genomic inflation factor lambda (λ) [11] was calculated for the BOLT-LMM and SNPTEST meta-analysis runs. This was complemented by generating quantile-quantile (QQ) [12] to investigate any early deviation of the expected P-values from the observed. Additionally, a Manhattan plot [12] was generated to highlight the BOLT-LMM index SNPs, and two more plots were created to mirror the BOLT-LMM signals with those from a GWAS of neutrophil count in people of African [13] and European [14] ancestry.

**Characterization of functional loci**

A query was placed through the variant effect predictor (VEP) [15] and FUMA [16] on the SNPs in the AFR_CAG filtered dataset. A further, broader literature search was conducted on the index and MR clumping SNPs using Ensembl [17], GeneCards [18], GWAS Catalog [19], The Human Protein Atlas [20], and the Genotype-Tissue Expression (GTEx) project [21].

**Heritability analysis**

An analysis was conducted with GCTA to estimate the proportion of variance in neutrophil count explained by all genetic variants present in the filtered AFR_CAG dataset [22]. First, a power calculation was done to assess whether the sample-size of unrelated people with neutrophil count data (N=5509) would be enough to detect genetic covariance [23]. Default power calculation parameters were used: α = 0.05, *h*^2^ = 0.3, var π = 2e-5; α = P-value significance threshold, *h*^2^ = combined genetic heritability for the trait, var π = variance of the off-diagonal elements of a genetic relationship matrix (GRM) [23]. Afterwards, a GRM was generated from the whole filtered AFR_CAG with the following command. A GRM is essentially a matrix with *n* rows and *y* columns, where n = number of individuals in sample and y = number of SNPs [24]. The *ny* matrix contains the minor allele counts for each SNPs of each individual [24], and it is used by LMM GWA software to adjust for population relatedness that can bias traditional linear model GWA analyses [25]. UKBB phenotypic data was then used to run GCTA-GREML, with and without adjusting for the Duffy SNP rs2814778. Yang et al. propose a way for estimating heritability while accounting for potential LD bias [26]. In brief, segment-based LD scoring was done on each chromosome. SNPs were stratified in R by LD scores in four groups for each chromosome [27], yielding 88 SNP groups in total. A GRM was generated for each SNP group, and GCTA-GREML was run similarly to the previous run.

**GWAS with additional covariates**

Several analyses were conducted to investigate and describe the phenotypic data in the AFR_CAG dataset. Descriptive statistics for neutrophil count were generated to provide information on the sample that the GWAS were run on. Missing data for additional variables were investigated, and an analysis was conducted to test whether missing data in each of these variables showed evidence of affecting neutrophil count. Moreover, a univariable, multivariable ANOVA type II and multivariable ANOVA type III were conducted to assess the variance explained by environmental, multifactorial and immutable (e.g. place of birth) variables. Following the results from the descriptive analyses, another GWAS was run in BOLT-LMM. “Genetic sex”, “time since last menstruation” and “menopause” variables were combined in a single discrete variable called “menstrual_status” and was created as follows: males, quartiles 1-4 of days since last menstruation, menopause, had hysterectomy. The covariates used in this run were sampling device, sample year, sample month, sample day, minutes passed in sample day, UN region of birth, K-means cluster, smoking status, alcohol drinker status, “menstrual_status”, age, body mass index and PCs 1 to 100. 669 individuals were filtered out for missing values and/or preferred not to answer in these variables, bringing the sample-size to 5,310.

**Description of working environment**

All analyses were performed in a Linux environment supported by the University of Bristol’s Advanced Computing Research Centre (ACRC) using the following publicly available software packages: PLINK v1.9 and v2.0 [28,29], QCTOOL v2.0.7 (https://www.well.ox.ac.uk/~gav/qctool/), LDSC v1.0.1 [30], SNPTEST v2.5.4 [2,3], BOLT-LMM v2.3.6 [31], META v1.7 [4], METAL v2011-03-25 [32–34], and GCTA v1.94.0 [5]. All other scripts, analyses, and figures were run and generated in the R environment using version 4.1.2 (Bird Hippie) [35] and Python environment using version 3.7.7 [36] on the ACRC computer clusters.

**References**

[1] Reich D, Nalls MA, Kao WHL, Akylbekova EL, Tandon A, Patterson N, et al. Reduced neutrophil count in people of African descent is due to a regulatory variant in the Duffy antigen receptor for chemokines gene. PLoS Genet 2009;5. https://doi.org/10.1371/journal.pgen.1000360.

[2] Marchini J, Howie B, Myers S, McVean G, Donnelly P. A new multipoint method for genome-wide association studies by imputation of genotypes. Nat Genet 2007 397 2007;39:906–13. https://doi.org/10.1038/ng2088.

[3] Burton PR, Clayton DG, Cardon LR, Craddock N, Deloukas P, Duncanson A, et al. Genome-wide association study of 14,000 cases of seven common diseases and 3,000 shared controls. Nature 2007;447:661. https://doi.org/10.1038/NATURE05911.

[4] de Bakker PIW, Ferreira MAR, Jia X, Neale BM, Raychaudhuri S, Voight BF. Practical aspects of imputation-driven meta-analysis of genome-wide association studies. Hum Mol Genet 2008;17:R122. https://doi.org/10.1093/HMG/DDN288.

[5] Yang J, Lee SH, Goddard ME, Visscher PM. GCTA: a tool for genome-wide complex trait analysis. Am J Hum Genet 2011;88:76–82. https://doi.org/10.1016/j.ajhg.2010.11.011.

[6] Yang J, Ferreira T, Morris AP, Medland SE, Genetic Investigation of AnTC, Consortium DiaIaGRAM (DIAGRAM), et al. Conditional and joint multiple-SNP analysis of GWAS summary statistics identifies additional variants influencing complex traits. Nat Genet 2012;44:369-S3. https://doi.org/10.1038/ng.2213.

[7] Marees AT, de Kluiver H, Stringer S, Vorspan F, Curis E, Marie-Claire C, et al. A tutorial on conducting genome-wide association studies: Quality control and statistical analysis. Int J Methods Psychiatr Res 2018;27:1–10. https://doi.org/10.1002/mpr.1608.

[8] Pruim RJ, Welch RP, Sanna S, Teslovich TM, Chines PS, Gliedt TP, et al. LocusZoom: regional visualization of genome-wide association scan results. Bioinforma Appl NOTE 2010;26:2336–7. https://doi.org/10.1093/bioinformatics/btq419.

[9] Boughton AP, Welch RP, Taliun D, Taliun G, Vandehaar P, Abecasis GR, et al. Interactive, shareable plots of GWAS data with LocusZoom n.d.

[10] Major T, Takei R. LocusZoom-like Plots for GWAS Results 2021. https://doi.org/10.5281/ZENODO.5154379.

[11] Yang J, Weedon MN, Purcell S, Lettre G, Estrada K, Willer CJ, et al. Genomic inflation factors under polygenic inheritance. Eur J Hum Genet 2011;19:807. https://doi.org/10.1038/EJHG.2011.39.

[12] Ehret GB. Genome-Wide Association Studies: Contribution of Genomics to Understanding Blood Pressure and Essential Hypertension. Curr Hypertens Rep 2010;12:17. https://doi.org/10.1007/S11906-009-0086-6.

[13] Chen MH, Raffield LM, Mousas A, Sakaue S, Huffman JE, Moscati A, et al. Trans-ethnic and Ancestry-Specific Blood-Cell Genetics in 746,667 Individuals from 5 Global Populations. Cell 2020;182:1198-1213.e14. https://doi.org/10.1016/j.cell.2020.06.045.

[14] Astle WJ, Elding H, Jiang T, Allen D, Ruklisa D, Mann AL, et al. The Allelic Landscape of Human Blood Cell Trait Variation and Links to Common Complex Disease. Cell 2016;167:1415-1429.e19. https://doi.org/10.1016/j.cell.2016.10.042.

[15] McLaren W, Gil L, Hunt SE, Riat HS, Ritchie GRS, Thormann A, et al. The Ensembl Variant Effect Predictor. Genome Biol 2016;17:1–14. https://doi.org/10.1186/S13059-016-0974-4/TABLES/8.

[16] Watanabe K, Taskesen E, Van Bochoven A, Posthuma D. Functional mapping and annotation of genetic associations with FUMA. Nat Commun 2017 81 2017;8:1–11. https://doi.org/10.1038/s41467-017-01261-5.

[17] Cunningham F, Allen JE, Allen J, Alvarez-Jarreta J, Amode MR, Armean IM, et al. Ensembl 2022. Nucleic Acids Res 2022;50:D988–95. https://doi.org/10.1093/NAR/GKAB1049.

[18] Safran M, Rosen N, Twik M, BarShir R, Iny Stein T, Dahary D, et al. The GeneCards Suite n.d. https://doi.org/10.1007/978-981-16-5812-9_2.

[19] Buniello A, Macarthur JAL, Cerezo M, Harris LW, Hayhurst J, Malangone C, et al. The NHGRI-EBI GWAS Catalog of published genome-wide association studies, targeted arrays and summary statistics 2019. Nucleic Acids Res 2019;47. https://doi.org/10.1093/NAR/GKY1120.

[20] Uhlén M, Fagerberg L, Hallström BM, Lindskog C, Oksvold P, Mardinoglu A, et al. Tissue-based map of the human proteome. Science 2015;347. https://doi.org/10.1126/SCIENCE.1260419.

[21] Lonsdale J, Thomas J, Salvatore M, Phillips R, Lo E, Shad S, et al. The Genotype-Tissue Expression (GTEx) project. Nat Genet 2013 456 2013;45:580–5. https://doi.org/10.1038/ng.2653.

[22] Yang J, Benyamin B, McEvoy BP, Gordon S, Henders AK, Nyholt DR, et al. Common SNPs explain a large proportion of heritability for human height. Nat Genet 2010;42:565. https://doi.org/10.1038/NG.608.

[23] Visscher PM, Hemani G, Vinkhuyzen AAE, Chen GB, Lee SH, Wray NR, et al. Statistical Power to Detect Genetic (Co)Variance of Complex Traits Using SNP Data in Unrelated Samples. PLOS Genet 2014;10:e1004269. https://doi.org/10.1371/JOURNAL.PGEN.1004269.

[24] VanRaden PM. Efficient Methods to Compute Genomic Predictions. J Dairy Sci 2008;91:4414–23. https://doi.org/10.3168/JDS.2007-0980.

[25] Loh P-R, Kichaev G, Gazal S, Schoech AP, Price AL. Mixed-model association for biobank-scale datasets. Nat Genet 2018 507 2018;50:906–8. https://doi.org/10.1038/s41588-018-0144-6.

[26] Yang J, Bakshi A, Zhu Z, Hemani G, Vinkhuyzen AAE, Lee SH, et al. Genetic variance estimation with imputed variants finds negligible missing heritability for human height and body mass index. Nat Genet 2015 4710 2015;47:1114–20. https://doi.org/10.1038/ng.3390.

[27] Evans LM, Tahmasbi R, Vrieze SI, Abecasis GR, Das S, Gazal S, et al. Comparison of methods that use whole genome data to estimate the heritability and genetic architecture of complex traits. Nat Genet 2018 505 2018;50:737–45. https://doi.org/10.1038/s41588-018-0108-x.

[28] Purcell S, Neale B, Todd-Brown K, Thomas L, Ferreira MAR, Bender D, et al. PLINK: A tool set for whole-genome association and population-based linkage analyses. Am J Hum Genet 2007;81:559–75. https://doi.org/10.1086/519795.

[29] Chang CC, Chow CC, Tellier LCAM, Vattikuti S, Purcell SM, Lee JJ. Second-generation PLINK: Rising to the challenge of larger and richer datasets. GigaScience 2015;4. https://doi.org/10.1186/s13742-015-0047-8.

[30] Bulik-Sullivan B, Loh PR, Finucane HK, Ripke S, Yang J, Patterson N, et al. LD Score Regression Distinguishes Confounding from Polygenicity in Genome-Wide Association Studies. Nat Genet 2015;47:291. https://doi.org/10.1038/NG.3211.

[31] Loh PR, Tucker G, Bulik-Sullivan BK, Vilhjálmsson BJ, Finucane HK, Salem RM, et al. Efficient Bayesian mixed-model analysis increases association power in large cohorts. Nat Genet 2015;47:284–90. https://doi.org/10.1038/ng.3190.

[32] Willer CJ, Li Y, Abecasis GR. METAL: Fast and efficient meta-analysis of genomewide association scans. Bioinformatics 2010;26:2190–1. https://doi.org/10.1093/BIOINFORMATICS/BTQ340.

[33] Sanna S, Jackson AU, Nagaraja R, Willer CJ, Chen WM, Bonnycastle LL, et al. Common variants in the GDF5-UQCC region are associated with variation in human height. Nat Genet 2008;40:198–203. https://doi.org/10.1038/NG.74.

[34] Willer CJ, Sanna S, Jackson AU, Scuteri A, Bonnycastle LL, Clarke R, et al. Newly identified loci that influence lipid concentrations and risk of coronary artery disease. Nat Genet 2008;40:161–9. https://doi.org/10.1038/NG.76.

[35] Core R Team. R: A Language and Environment for Statistical Computing. R Found Stat Comput 2019;2:https://www.R--project.org. http://www.r-project.org (accessed March 2, 2021).

[36] The Python Language Reference — Python 3.7.13 documentation n.d. https://docs.python.org/3.7/reference/ (accessed August 5, 2022).
