# Supplementary material for: A genome-wide association study of neutrophil count in individuals associated to an African continental ancestry group facilitates studies of malaria pathogenesis
Source: Hum Genomics. 2024 Mar 15;18:26. doi: 10.1186/s40246-024-00585-w (PMC10941368; doi:10.1186/s40246-024-00585-w)
Supplement: Supplementary file 2 — Additional file 2. Supplementary Figures S1–S13. [file 40246_2024_585_MOESM2_ESM.docx]

# SUPPLEMENTARY FIGURES

**Figure S1**: Neutrophil count variation in the GWAS sample.


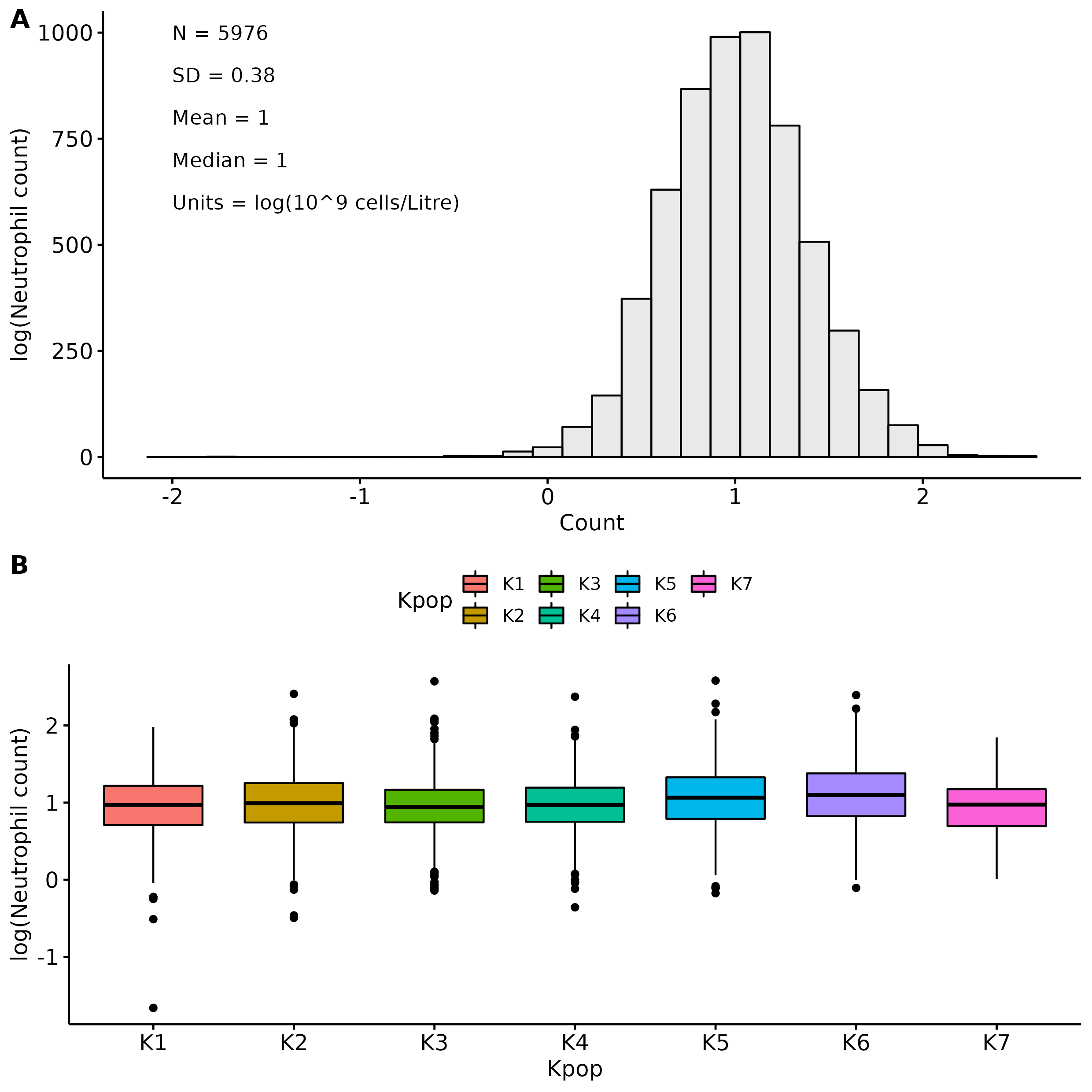


**Figure S1.** A histogram outlining the distribution of neutrophil levels is shown in the whole AFR_CAG population (A), along with representative boxplots describing neutrophil count variation by K-means cluster sample (B).

## **Figure S2**: Power calculation.


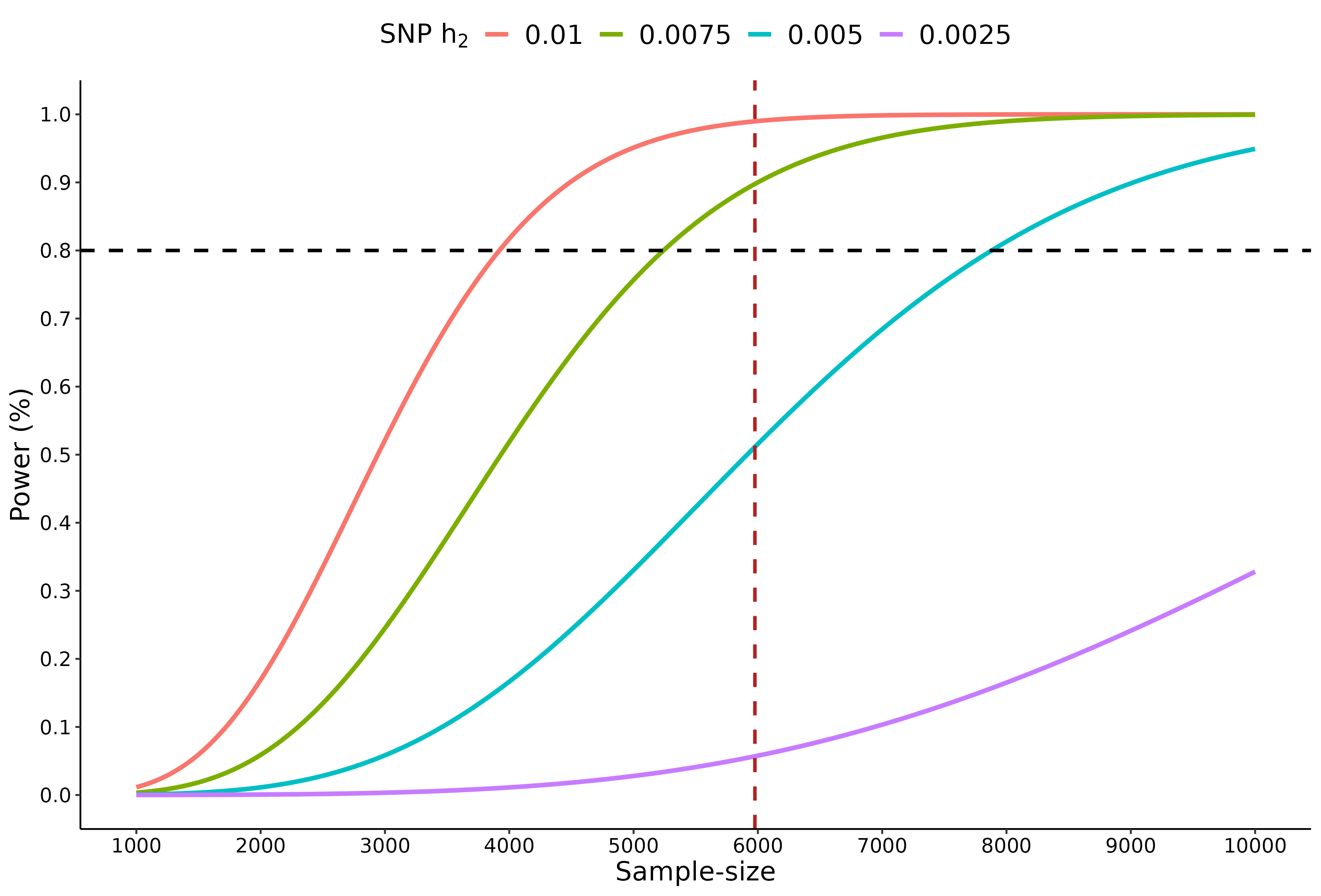


**Figure S2.** Power calculation of a GWAS AFR_CAG sample. The x-axis indicates the sample-size, while the y-axis is the statistical power of an association test. Each curved line shows how power varies by sample-size at different degrees of the variance explained by all the SNPs on neutrophil count (1%, 0.75%, 0.5%, 0.25%). A black horizontal line is fixed at Power=80%, and a red vertical line is drawn at the GWAS sample size of 5,976.

## **Figure S3**. Regional plots of index SNPs (1).


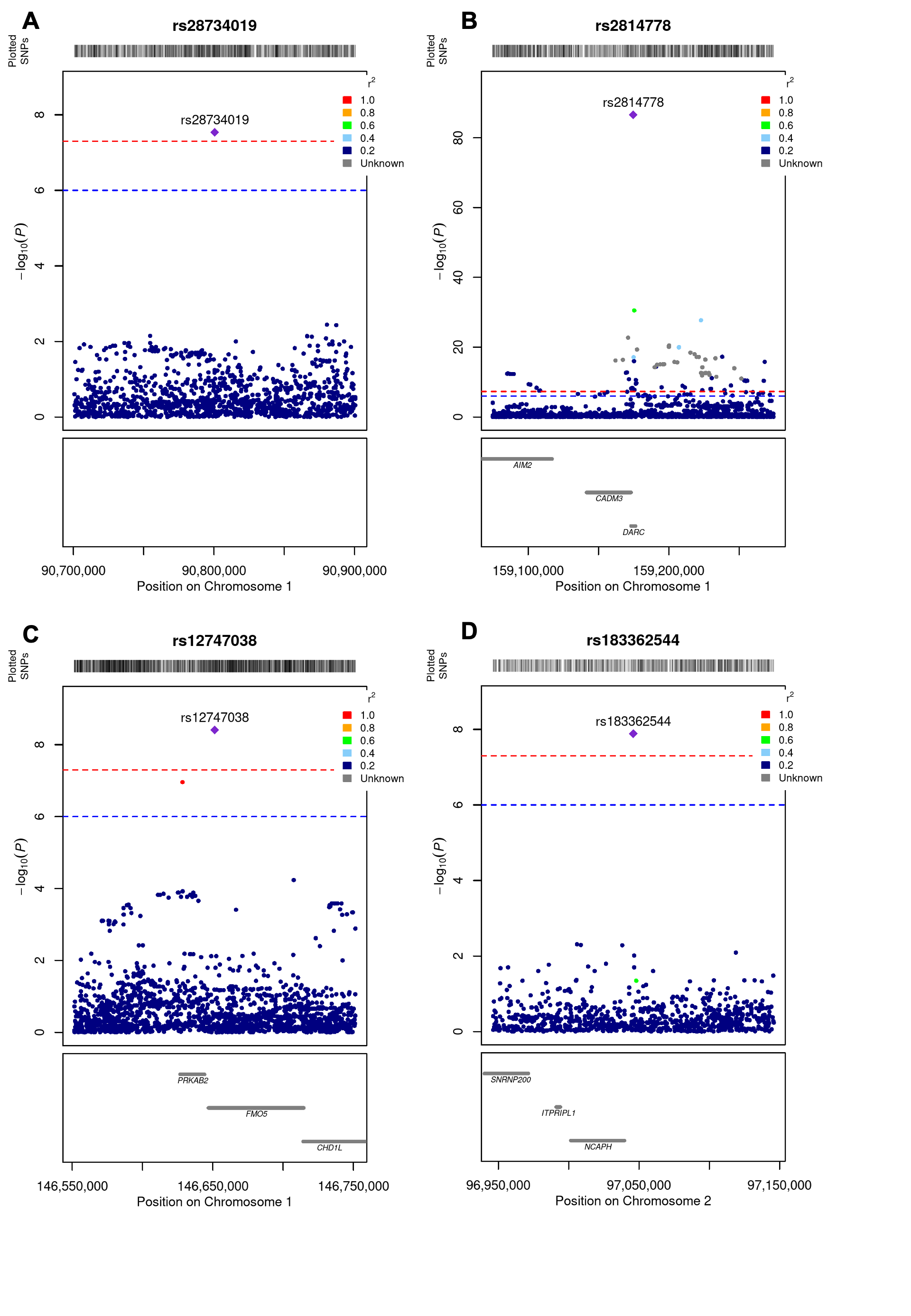


## **Figure S4**. Regional plots of index SNPs (2).


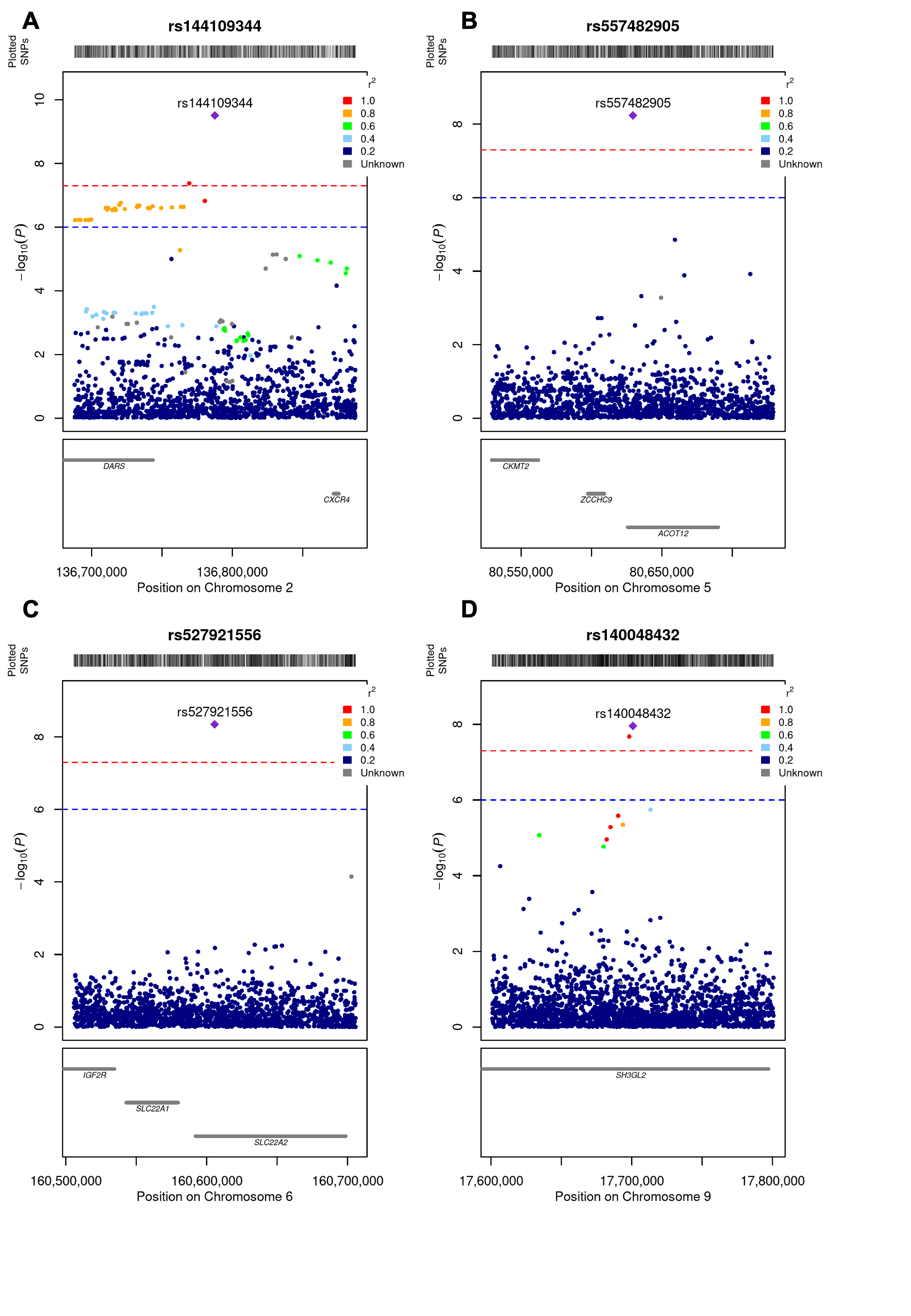


## **Figure S5**. Regional plots of index SNPs (3).


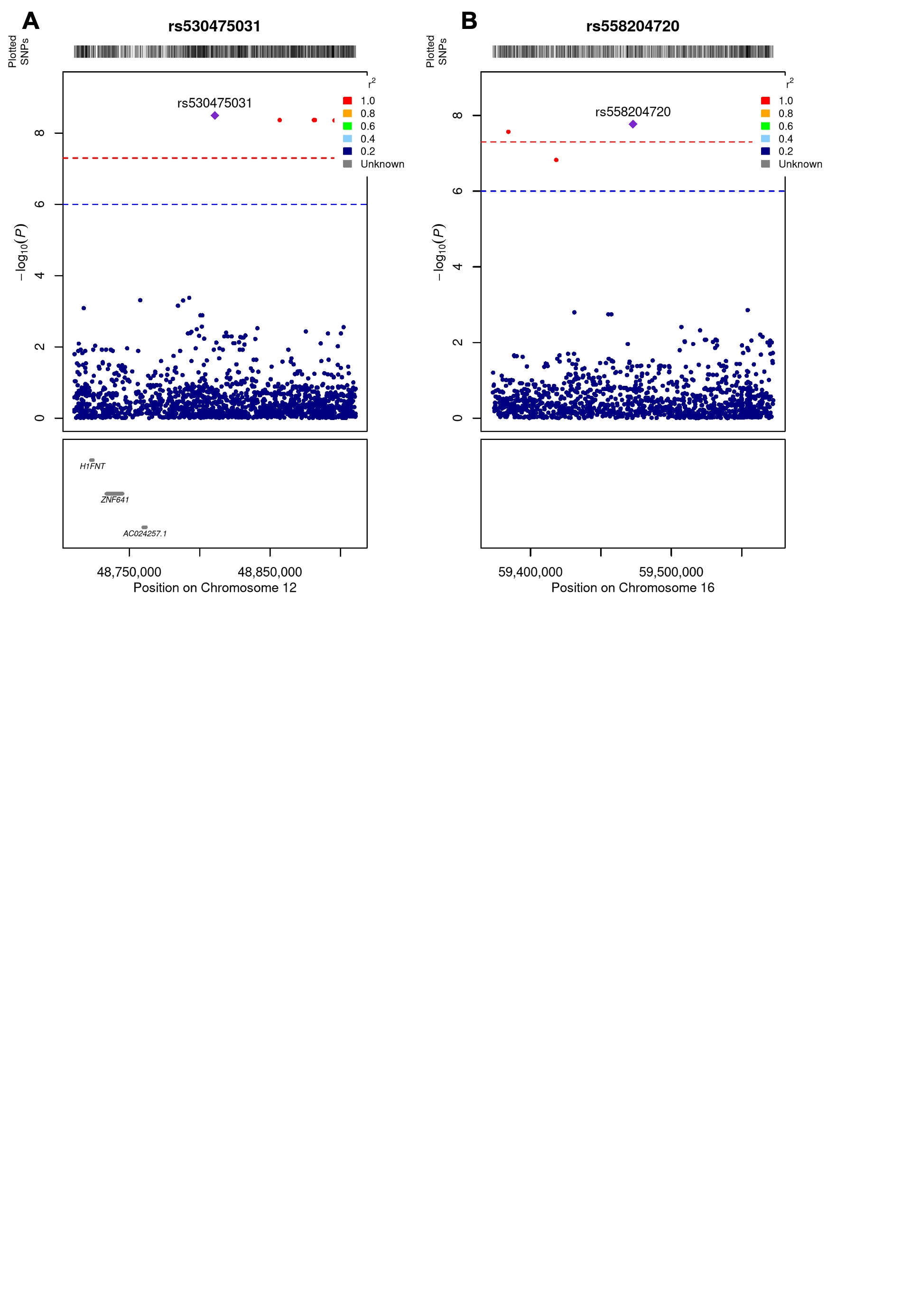


## **Figure S6**: Forest plot of index SNPs effect sizes.


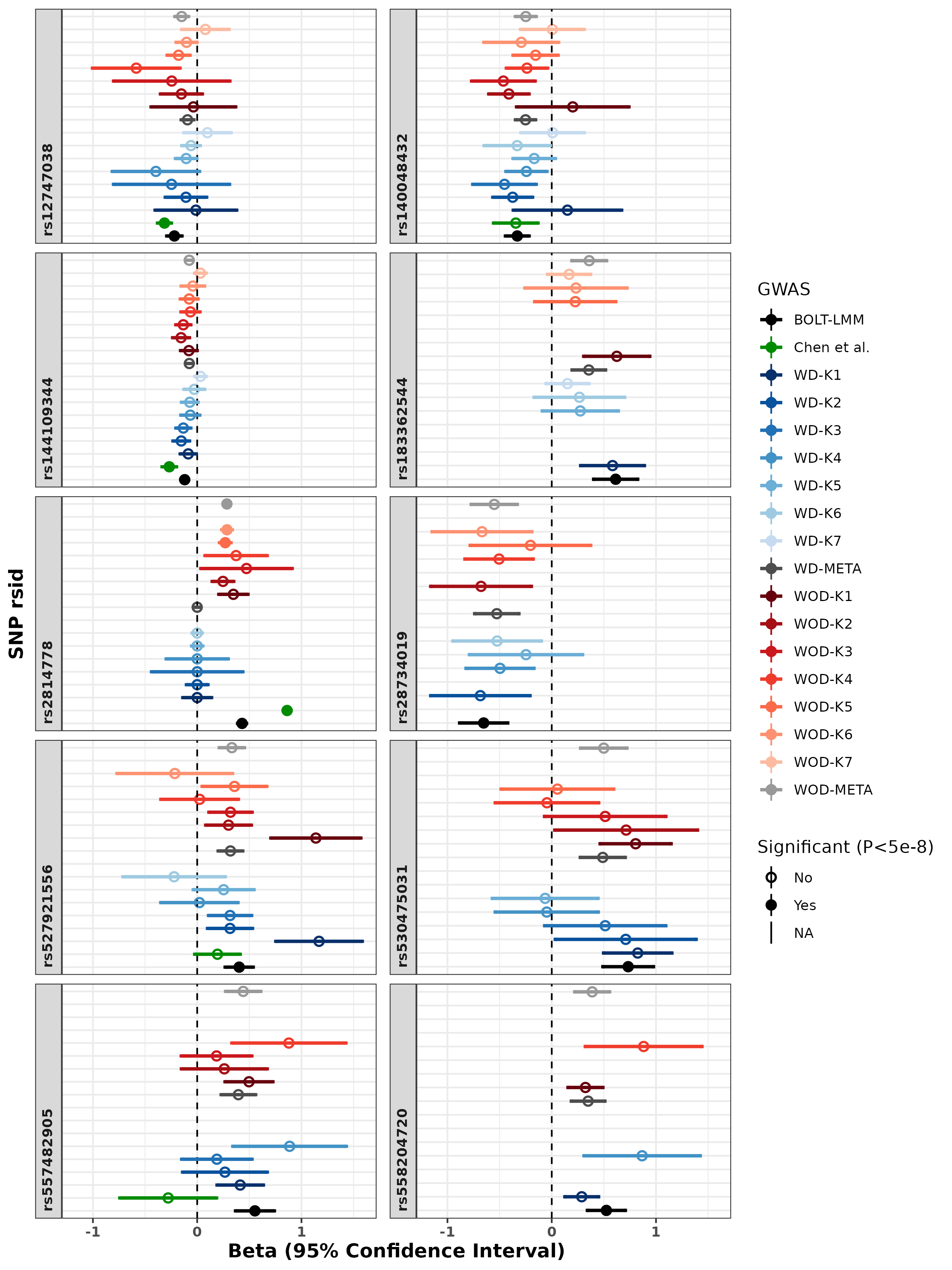


**Figure S6.** Forest plot of index SNPs by K-means cluster. The effect-size of each BOLT index SNP was compared to that from SNPTEST/META, by-Kpop runs and Chen et al GWAS. Effect-sizes for each SNP across GWAS are present in the respective boxes of the figure. The x-axis indicates the effect-size (beta coefficient) of each SNP with 95% CIs, while the y-axis is the type of GWAS (indicated by the figure legend colouring). Some effect sizes were not displayed, either due to a low minor allele count in the case of the Kpop GWAS, or due to not being present in the summary statistics, in the case of the Chen GWAS. Non-signif. WD = adjusting for rs2814778; WOD = without adjusting for rs2814778.

## **Figure S7**. Scatter plot of GCTA-COJO effect sizes.


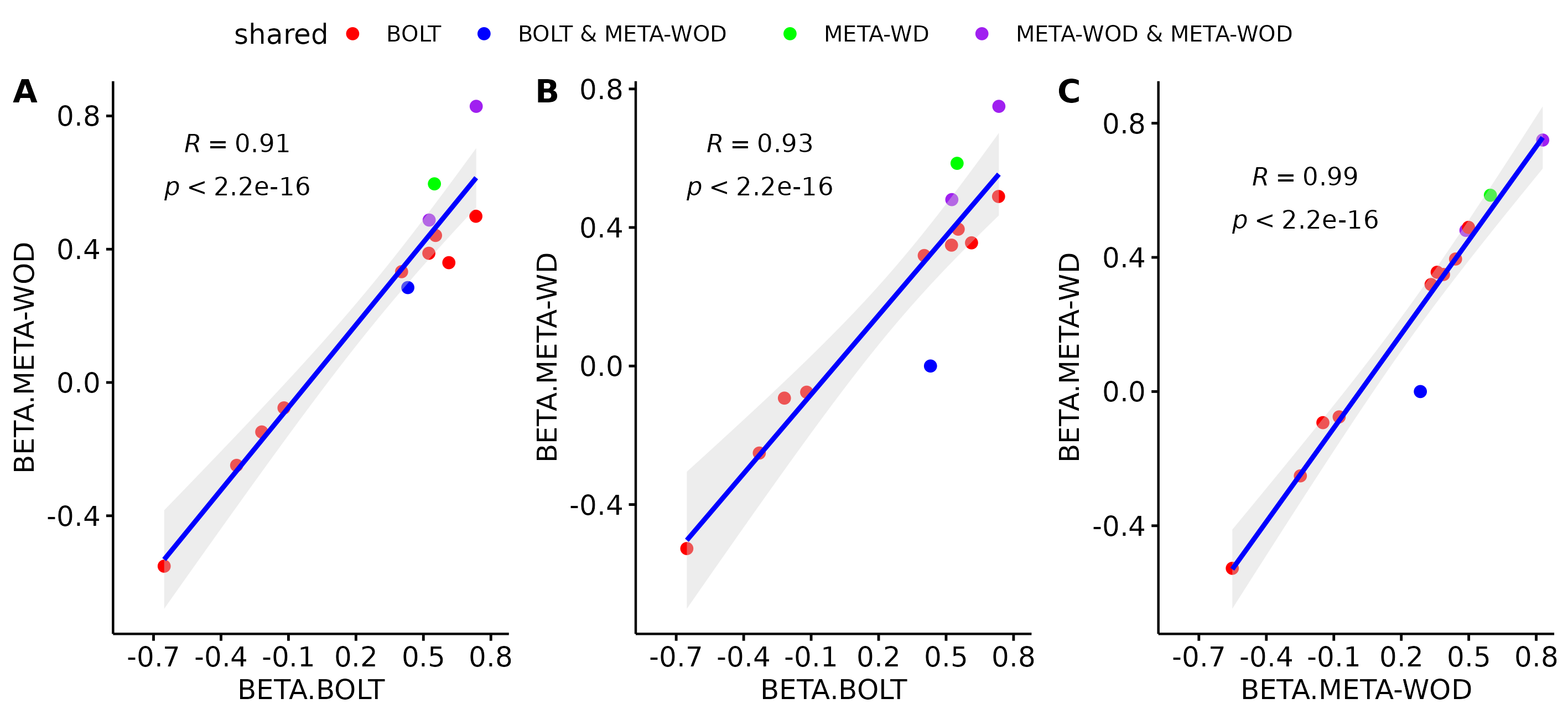


**Figure S7.** Scatter plot of GCTA-COJO effect sizes. Comparison of effect sizes of all GCTA-COJO independent signals of BOLT-LMM with META-WOD (A), BOLT-LMM with META-WD (B) and META-WOD with META-WD (C).

## **Figure S8**: Description of genomic risk loci.


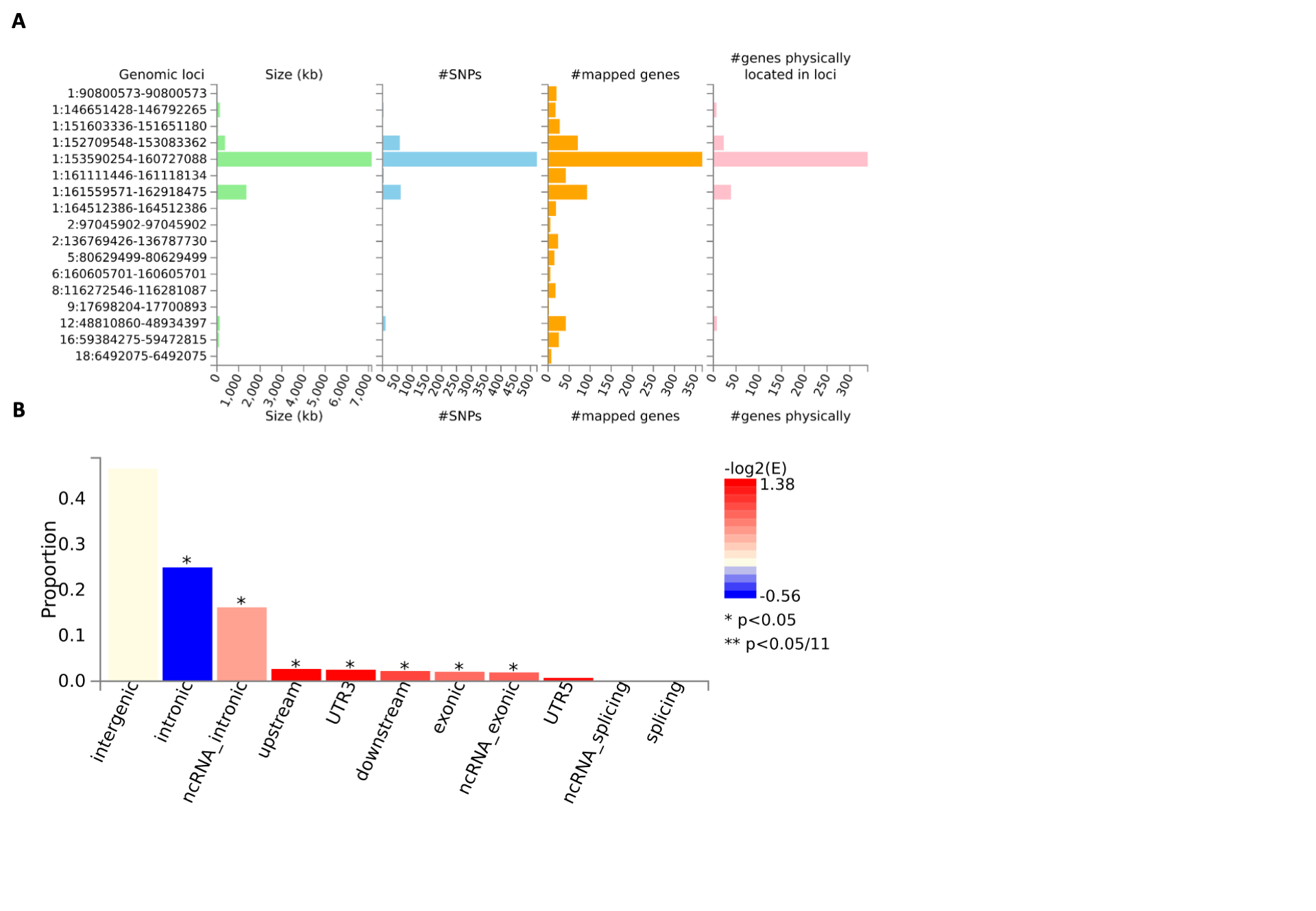


**Figure S8.** Description of genomic risk loci. FUMA analysis results for SNPs passing the GWAS significance threshold in the BOLT-LMM filtered GWAS.


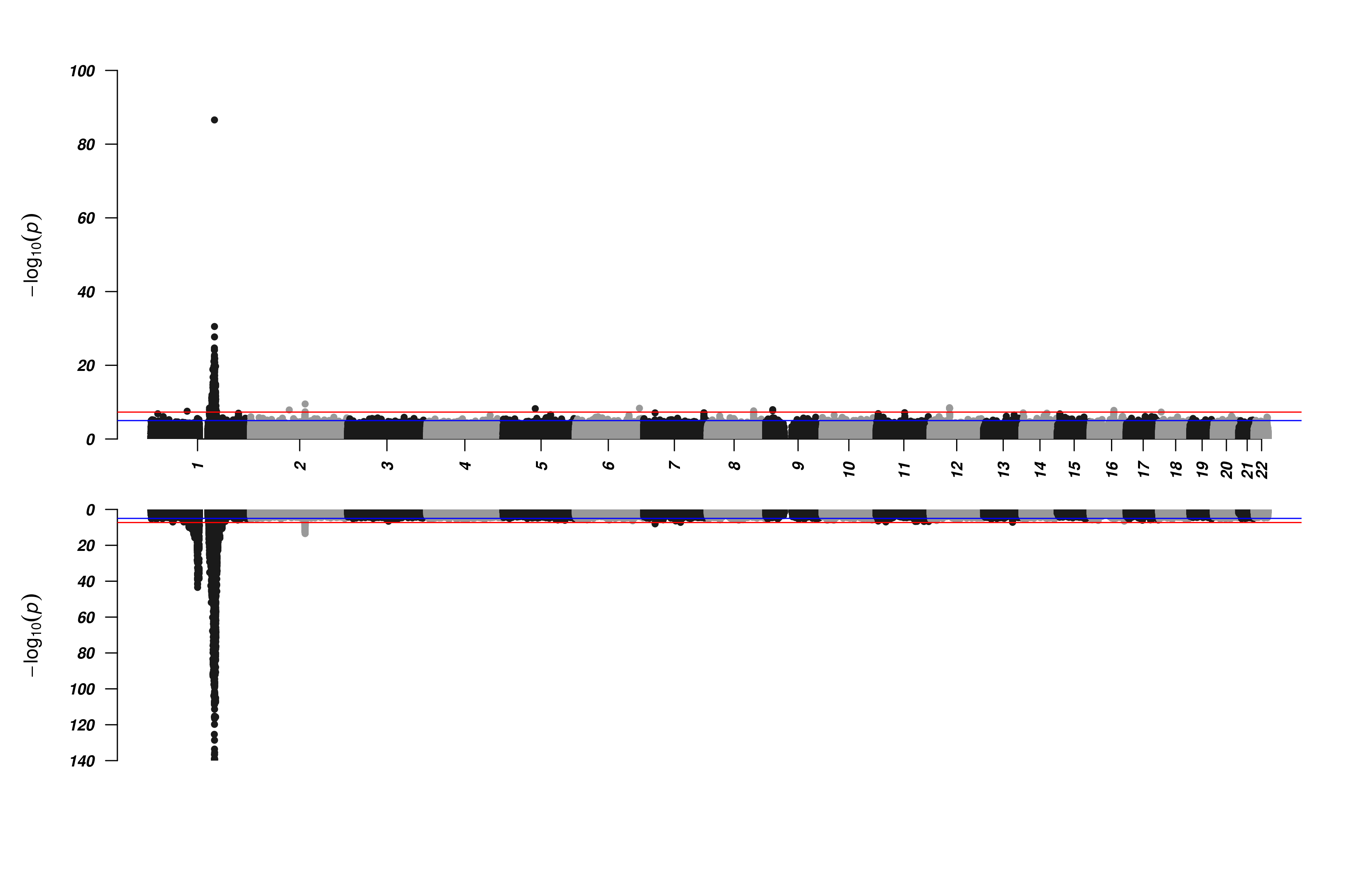


## **Figure S9**. Comparison of GWAS results for neutrophil count in Africans. Manhattan plot of BOLT-LMM neutrophil count GWAS from our study (top) mirrored with another Manhattan plot generated using summary statistics from a GWAS of neutrophil count done in people of African ancestry (Chen et al, reference).


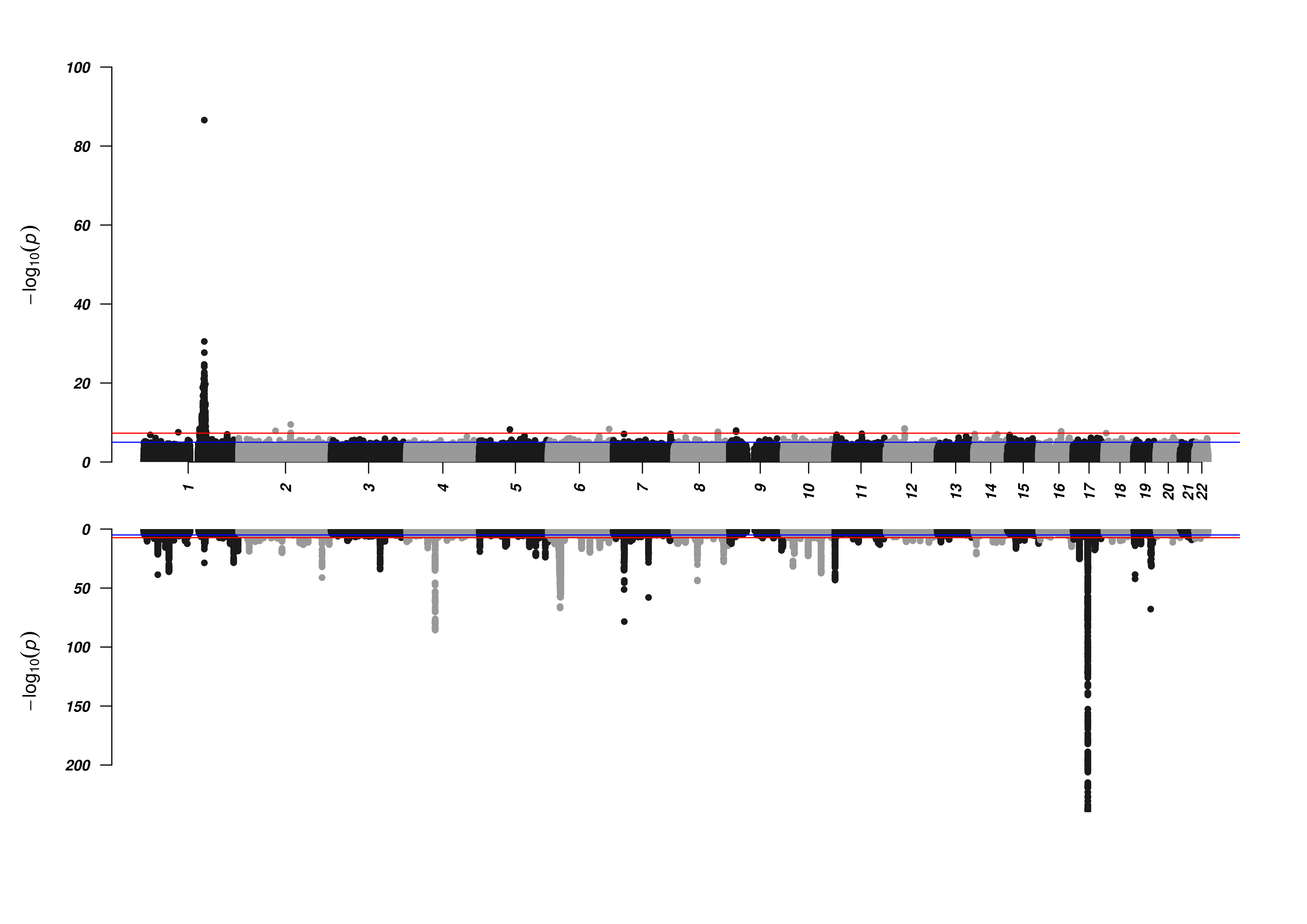


**Figure S10.** Comparison of GWAS results for neutrophil count in Europeans. Manhattan plot of BOLT-LMM neutrophil count GWAS from our study (top) mirrored with another Manhattan plot generated a GWAS of neutrophil count done in people of European ancestry in UK Biobank by Astle et al 10.1016/j.cell.2016.10.042.

##
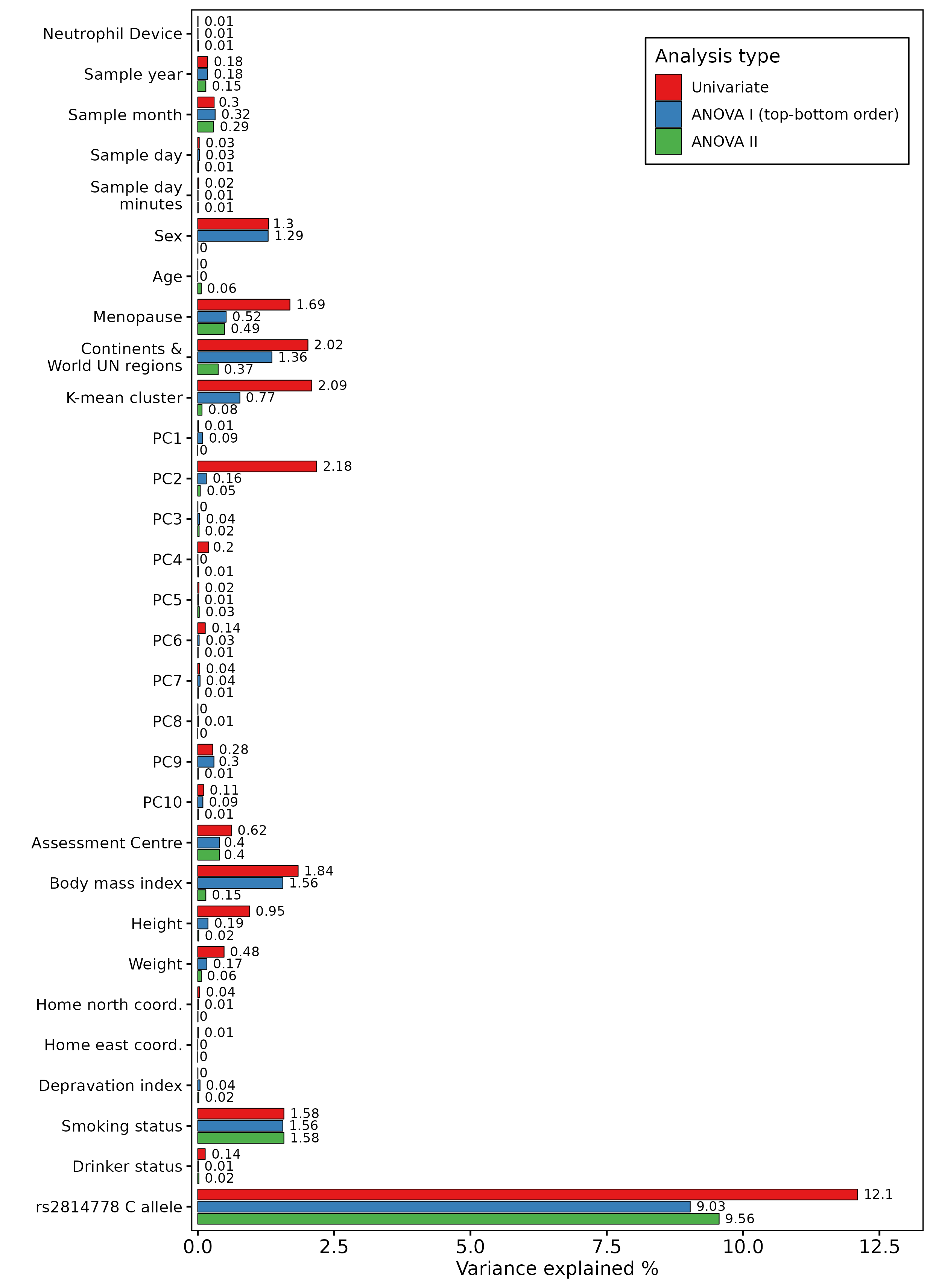


## **Figure S11**: Proportion of variance explained by traits on neutrophil count. The x-axis indicates the trait studied, while the y-axis indicates the proportion of variance explained (PVE) on neutrophil count by that trait. The PVE of each trait was studied in a univariable manner (red), adjusted for only the other traits above in an ANOVA I hierarchical manner (blue), and adjusted between all other traits in a ANOVA II manner.

##
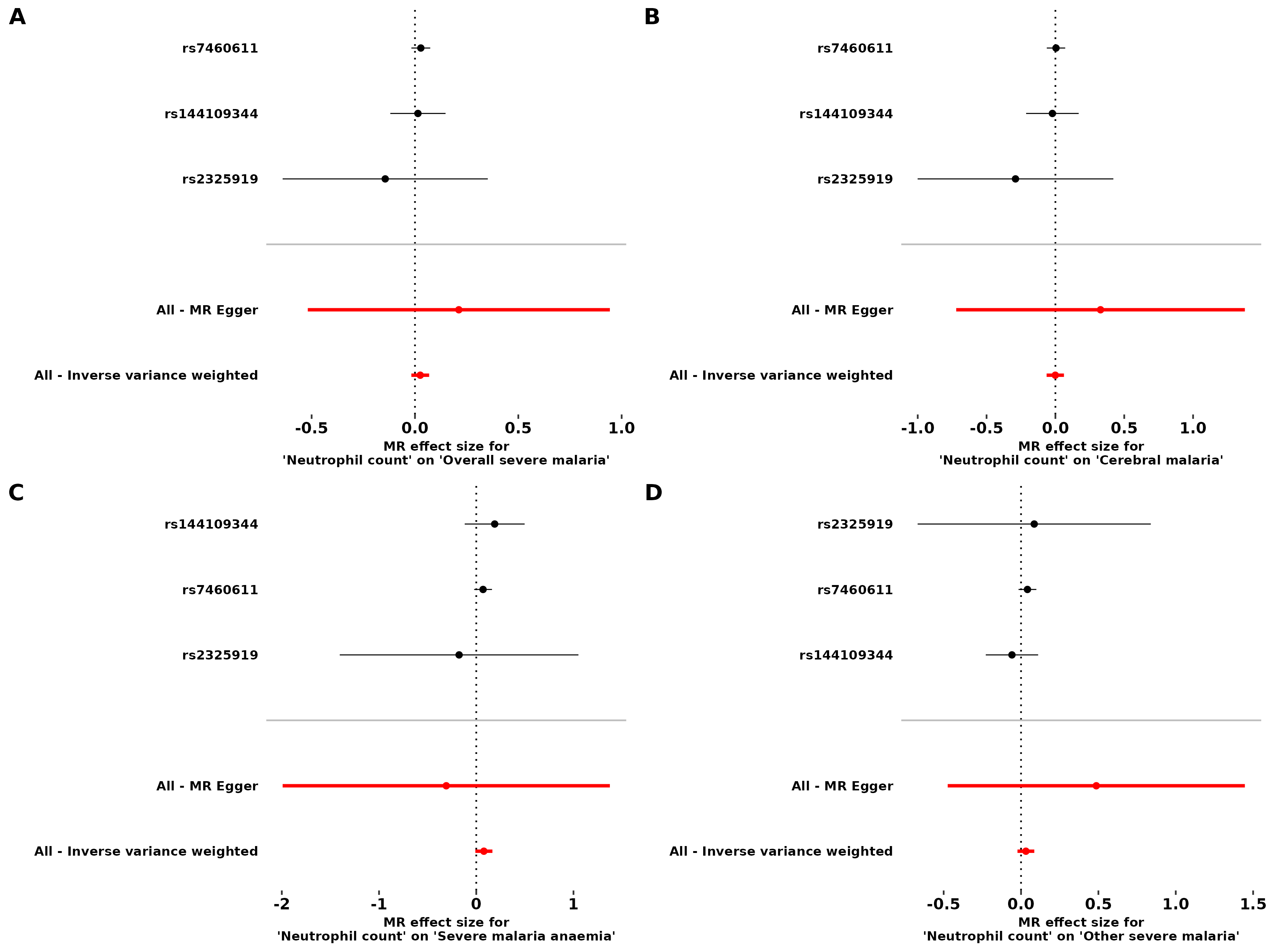


## **Figure S12**: Single-SNP MR analysis of neutrophil count on severe malaria and its subtypes. SNPs proxying for neutrophil count are shown on the x-axis. The effect of each SNP proxying for neutrophil count is displayed on the y-axis, along with 95% CIs. The MR-Egger and IVW MR methods are shown below the single-SNP analysis.

##
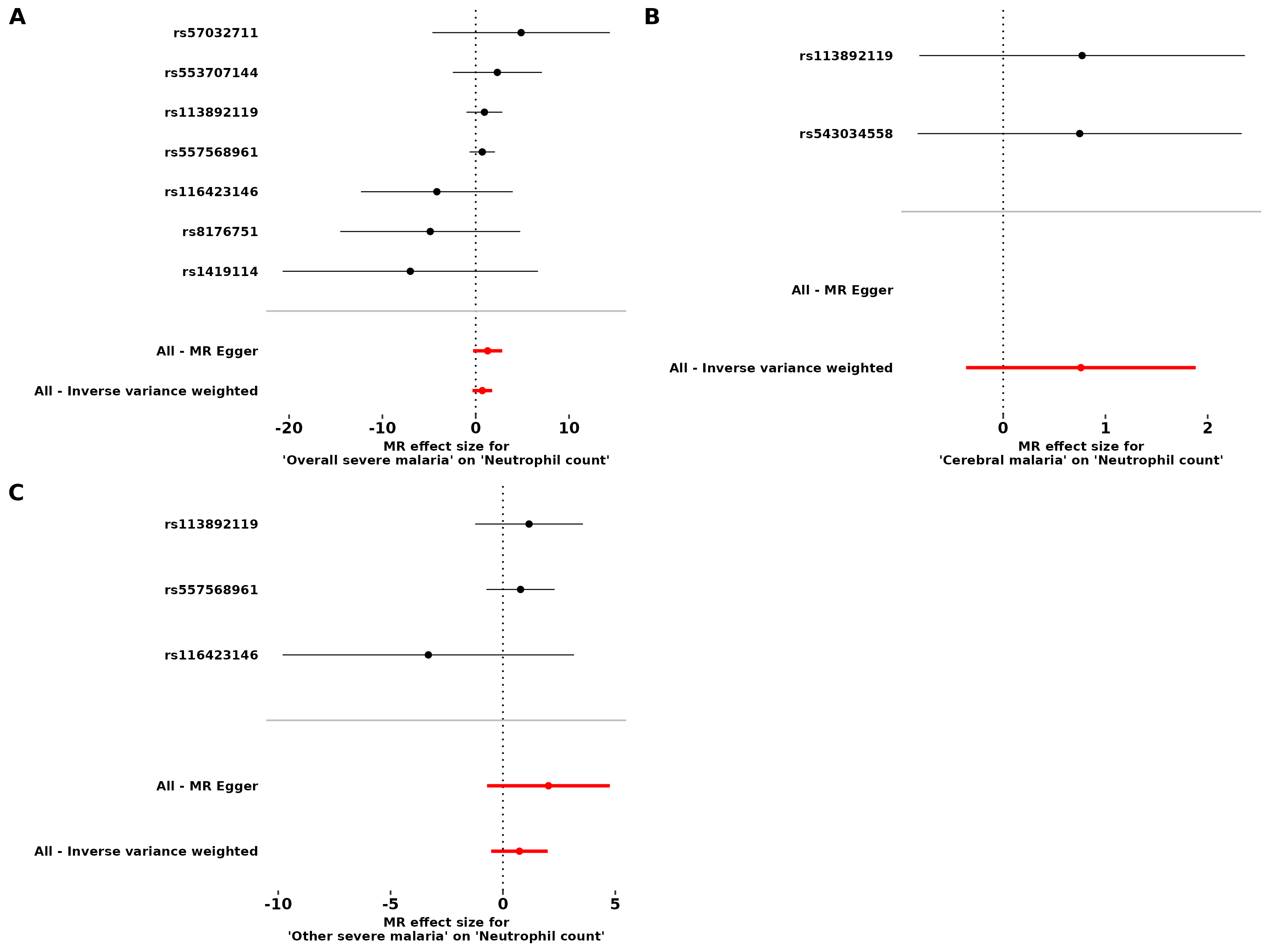


## **Figure S13:** Single-SNP MR analysis of severe malaria on neutrophil count. SNPs proxying for liability to severe malaria are shown on the x-axis. The effect of each SNP proxying for liability to severe malaria is displayed on the y-axis, along with 95% CIs. The MR-Egger and IVW MR methods are shown below the single-SNP analysis.
